# Freestyle master’s swimming: Nationality, sex, and performance trends in World Aquatics competitions (1986–2024)

**DOI:** 10.1371/journal.pone.0332040

**Published:** 2025-09-10

**Authors:** Wais Ahmad, Matthias Wilhelm, Sascha Moreitz, Marilia Santos Andrade, Pedro Forte, Arkadiusz Stanula, Pantelis T. Nikolaidis, Ivan Cuk, Mabliny Thuany, Katja Weiss, Thomas Rosemann, Lee Hill, Aldo Seffrin, Beat Knechtle

**Affiliations:** 1 Centre for Rehabilitation & Sports Medicine, Inselspital Bern, University Hospital Bern, Bern, Switzerland; 2 Department of Radiology, Lucerne Cantonal Hospital, Lucerne, Switzerland; 3 Department of Physiology, Federal University of São Paulo, São Paulo, Brazil; 4 CI-ISCE, Higher Institute of Educational Sciences of the Douro, Penafiel, Portugal; 5 Department of Sports Sciences, Instituto Politécnico de Bragança, Bragança, Portugal; 6 Research Center for Active Living and Wellbeing (Livewell), Bragança, Portugal; 7 Department of Swimming and Water Rescue, Institute of Sport Sciences, Academy of Physical Education in Katowice, Katowice, Poland; 8 School of Health and Caring Sciences, University of West Attica, Athens, Greece; 9 Faculty of Sport and Physical Education, University of Belgrade, Belgrade, Serbia; 10 Faculty of Sport, University of Porto, Porto, Portugal; 11 Institute of Primary Care, University Hospital of Zurich, Zurich, Switzerland; 12 Department of Pediatric Surgery, Research Institute of the McGill University Health Centre, Montreal, Canada; 13 Postgraduate Program in Translation Medicine, Federal University of São Paulo, São Paulo, Brazil; 14 Medbase St. Gallen Am Vadianplatz, St. Gallen, Switzerland; Università degli Studi di Milano: Universita degli Studi di Milano, ITALY

## Abstract

**Background:**

In sports science, freestyle swimming has been thoroughly studied for particular performance-related factors. Nonetheless, it is unknown what countries the top freestyle swimmers are from, especially not for age group swimmers. In addition, the existing research on the performance of master freestyle swimmers has yet to confirm that male swimmers achieve faster times than their female counterparts across all age groups and distances. The current study looked into the nationalities and sexes of the top freestyle swimmers in each age group in World Aquatics competitions for the 50m, 100m, 200m, 400m, and 800m events from 1986 to 2024.

**Methods:**

The data (derived from (www.worldaquatics.com/masters/archives/masters-archives) were presented using mean, standard deviation, maximum and minimum values, and/or confidence intervals. The year of competition, age, age group, stroke, distance, and first and last names of each swimmer were noted. The nations were then divided into six groups: one group comprising all other countries and the top five nations with the greatest number of appearances in the top 10 fastest freestyle swimming times by distance each year.

**Results:**

In freestyle swimming, most swimmers (30.6%) competed in the 50m event (n = 25,094, 10,909 female and 14,185 male), followed by the 100m event (25.6%, n = 20,961, 8,796 female and 12,165 male), the 200m event (17.4%, n = 14,309, 6,729 female and 7,580 male), the 400m event (13.4%, n = 10,956, 5,363 female and 5,593 male), and the 800m event (12.6%, n = 10,317, 5,179 female and 5,138 male). The results from the generalized linear models indicate that sex, age group, and the interaction between sex and age group all had significant effects on the 50m, 100m, 200m, 400m, and 800m races. Specifically, for the 50m races, the effect of sex was significant (x^2^ (1) = 3451.941, p < 0.001), as was the effect of age group (χ^2^ (13) = 19,295.169, p < 0.001), and the interaction between sex and age group (χ ^2^ (13) = 654.671, p < 0.001).

**Conclusion:**

The USA demonstrates quantitative dominance by contributing the greatest number of top 10 performers. Additionally, the study highlights significant sex-based performance differences, with males generally outperforming females in all age categories. This study comprehensively analyzes the performance trends observed in freestyle master swimming for nearly four decades.

## Introduction

Over the last few decades, master’s swimming has been one of the most successful master’s athletic organizations [1]. As a special class of competitive swimming for swimmers 25 years and older (i.e., age groups 25–29–90–94 years), the master‘s program became part of the “Fédération Internationale de Natation” (FINA), now known as World Aquatics, World Championships, debuting in 1986 at Tokyo, Japan [2]. World Aquatics serves as the international governing body for aquatic sports, including masters swimming. Apart from the Olympic Games and the World Championships for elite swimmers, World Aquatics holds the World Master’s Championships for all disciplines and distances in open-water and pool swimming [3].

Swimming as a sports discipline can be performed in various styles, usually called “strokes” [4–7,8]. Freestyle swimming – one of the fundamental strokes within competitive swimming – is specifically characterized by a continuous alternating arm and flutter-kicking motion [4–7,9]. Though many different facets of freestyle swimming have been studied, such as its physiological (i.e., energy cost, oxygen uptake) [10], biomechanical (i.e., kinetic and kinematics) [11,10], and psychological (i.e., mood, stress, anxiety and motivation) [12,13–15] aspects, there exists a notable research gap in understanding the influence of nationality on participation and performance dynamics among age group freestyle swimmers [16,9]. While studies have delved into factors such as sex differences [17,18] and trends in participation and performance [8] among freestyle master swimmers, the interplay between nationality, sex, performance trends, and its impact on these dynamics remains relatively unexplored. Though it has been studied explicitly in the open-water swimming field [19,20–22], little is known about swimming distances.

Regarding nationality, the USA’s dominance of top swimmers is well documented [23,24]. Firstly, the US has a successful history in international swimming competitions, including the Olympics, Pan Pacific Championships, and World Championships. The American swimmers have consistently dominated the medal standings, demonstrating the country’s ability to produce top swimmers [9,25]. Secondly, the US has a significant swimming infrastructure, with many clubs and programs, resulting in a larger pool of talented swimmers from which top athletes emerge [26,27].

Further analysis of past performance data in various age group categories revealed that male swimmers have exhibited consistently faster swimming times than female swimmers [6,7,17,28–31]. Declines in performance with age have also been documented in longitudinal cohorts of masters’ champions [32]. Male’s physiological advantages (e.g., greater muscle mass and lung capacity compared to females in the same age group) can translate to superior performance in swimming [17,33]. For example, a study by Knechtle et al. [34] showed that when examining race times in 3000m freestyle, male swimmers consistently outperformed female across almost all age groups. However, evidence also indicates that female swimmers have been narrowing the gap with male in open-water swimming, particularly in long-distance events where the assumption of outperforming male was previously held [8]. Additionally, males are prone to have higher participation numbers in short-distance competitions [35], whereas females tend to show higher similar participation numbers in long distances [36,37]. This analysis of male’s and female’s participation at different distances and sports has been evaluated in previous studies, especially in individual sports [17,38–40]. However, the variance of the sex ratio participation between 50m and 800m is not clear.

As it is unclear where the best master freestyle swimmers in each age group and sex come from geographically, the goal of this study is to look at trends in master swimmers’ participation and performance in freestyle 50m, 100m, 200m, 400m and 800m events held in World Aquatics competitions between 1986 and 2024, broken down by age groups, sex, and nationality. Based on previous research, we hypothesized that for freestyle swimming: (i) the USA would demonstrate quantitative dominance by contributing the greatest number of top 10 performers, and we subsequently sought to determine if this was matched by qualitative dominance in the form of superior average performance times; (ii) males would outperform females in the same age category; (iii) the females tend to equalize the male’s participation number in long distances.

## Methods

### Ethical approval

The Institutional Review Board of Kanton St. Gallen, Switzerland, reviewed and approved this study (EKSG 01/06/2010), granting a waiver for participant informed consent due to the use of publicly available data. The research was carried out in compliance with the ethical principles outlined in the Declaration of Helsinki, originally established in 1964 and updated in 2013.

### Data set and data preparation

The race data were sourced from the official World Aquatics website (www.worldaquatics.com). Full data was obtained from all World Masters Championships held between 1986 and 2024 (www.worldaquatics.com/masters/archives/masters-archives). For each swimmer, the year of competition, first name, last name, age, age group, stroke, and distance were recorded. Nationalities were subsequently classified into six categories: the five nationalities having the highest frequency of appearances in the top 10 fastest times in freestyle swimming by distance each year, together with a collective group of all other nationalities. The top 10 times for 1988 were excluded from the nationality analysis due to the lack of available information in the database. Furthermore, no male’s data was available for 2008. No competitions occurred during the pandemic from 2020 to 2022; therefore, no data is available. All competition data analyzed were from events held in long course meter (LCM) 50-meter pools. It should be noted that the swimmers from Russia were not allowed to compete under their flag in 2023 and 2024 for political reasons. Their nationality was abbreviated to “NIA”. However, as they are included in the statistics from 1986 to 2019, their nationality was changed from “NIA” to “RUS”.

### Statistical analysis

The mean, standard deviation, maximum and minimum values, and/or confidence intervals were used to display descriptive data. For descriptive purposes, the top 10 race times for each sex and swimming distance were determined. Following that, nationalities were divided into six groups: one group that included all other nationalities and the top five that appeared in the freestyle swimming top 10 times annually by distance. Additionally, for descriptive purposes as presented in Table 1, the ratio of female to male competitors within each age group and swimming distance was calculated (number of females/ number of males) to illustrate the participation balance between sexes. Shapiro-Wilk and Levene’s tests revealed that the data did not have homogeneous variances or a normal distribution. Therefore, multiple pairwise comparisons modified by Bonferroni correction were carried out to discover differences, and the Kruskal-Wallis H test was employed to compare differences in the mean race times of the top 10 swimmers between the different nations. The impact of age group and sex on swimming time was evaluated using generalized linear models (GLMs) with a log link function and gamma probability distribution. The post hoc Bonferroni test was used to examine any differences that were discovered, and possible interactions with sex and age group were also examined. Based on the model linking function’s lowest value, the dependent variable’s distribution was selected using the Akaike Information Criterion (AIC) [41]. To make sure the model performs better than the null model, the Omnibus test was also employed. Statistical analyses were performed using SPSS version 26.0 (SPSS, Inc., Chicago, IL, USA), with a significance level set at 0.05.

**Table 1:** Ratio of female to male by age and distance.

	50m			100m			200m			400m			800m		
Age group	Female	Male	Ratio	Female	Male	Ratio	Female	Male	Ratio	Female	Male	Ratio	Female	Male	Ratio
25-29	1033	1494	0.69	977	1431	0.8	660	710	0.93	467	432	1.08	392	358	1.09
30-34	1101	1587	0.69	973	1496	0.65	666	845	0.79	549	596	0.92	472	442	1.07
35-39	1075	1673	0.64	942	1441	0.65	729	837	0.87	535	635	0.84	529	521	1.02
40-44	1278	1881	0.68	1003	1538	0.65	702	919	0.76	611	653	0.94	564	543	1.04
45-49	1290	1769	0.73	1001	1457	0.69	794	869	0.91	577	690	0.84	623	602	1.03
50-54	1266	1508	0.84	995	1336	0.74	786	804	0.98	647	605	1.07	649	614	1.06
55-59	1100	1133	0.97	834	990	0.84	636	751	0.85	531	530	1.00	558	520	1.07
60-64	1010	1082	0.93	703	840	0.84	574	604	0.95	488	443	1.10	468	496	0.94
65-69	728	774	0.94	558	596	0.94	453	457	0.99	379	368	1.03	363	393	0.92
70-74	499	553	0.90	357	477	0.75	313	344	0.91	289	282	1.02	291	299	0.97
75-79	284	370	0.77	254	299	0.85	191	245	0.78	177	191	0.93	166	204	0.81
80-84	167	222	0.75	132	151	0.87	120	126	0.95	75	109	0.69	65	105	0.62
85-89	51	90	0.57	47	75	0.63	46	49	0.94	27	48	0.56	25	37	0.68
90+	27	49	0.55	20	38	0.53	19	20	0.95	11	11	1.00	14	4	3.50

## Results

A total of 204,005 swimmers (94,312 female and 109,693 male) competed in 50m, 100m, 200m, 400m, and 800m freestyle races between 1986 and 2024. The majority of swimmers (40.2%) competed in freestyle events (n = 81,597; female = 36,936; male = 44,661 male). Most swimmers (30.8%) competed in the 50m event (n = 25,094; female = 10,909; male = 14,185), followed by the 100m event (25.7%; n = 20,961; female = 8,796; male = 12,165), the 200m event (17.5%; n = 14,269; female = 6,689; male = 7,580), the 400m event (13.4%; n = 10,956; female = 5,363; male = 5,593), and the 800m event (12.6%; n = 10,317; female = 5,179; male = 5,138). The ratio of female to male competitors in each swim distance and age group in freestyle events was calculated for descriptive analysis, as illustrated in Table 1.

**Fig 1:**
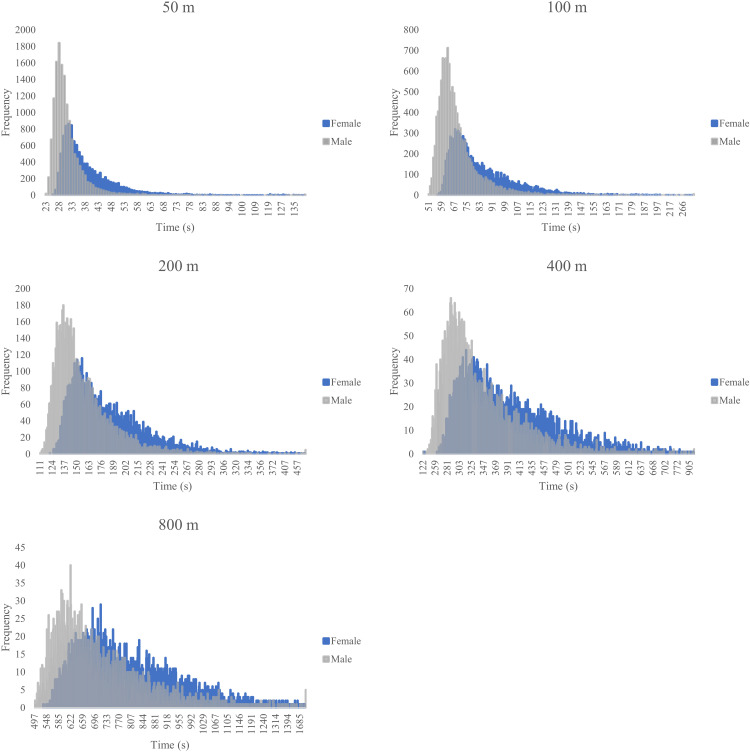
Histograms of race times for female and male swimmers across all freestyle swimming distances.

As a general overview, the mean race times aggregated across all age groups showed expected increases with distance and differences between sexes (e.g., 50m females: 39.16 ± 11.74 s, males: 31.02 ± 6.48 s; 800m females: 806.40 ± 174.77 s, males: 707.62 ± 172.03 s). Specific minimum and maximum times reflect the wide range within the master swimmer population. Performance variations across specific age groups, which provide more detailed insights, are analyzed further below (see Fig 3). The 50m freestyle event had an overall mean time of 34.56 ± 10.0 seconds (minimum 22.94/ maximum 156.18); females had a mean time of 39.16 ± 11.74 (minimum 25.73/ maximum 156.18) and males 31.02 ± 6.48 (minimum 22.94/ maximum 137.68). The 100m freestyle event had an overall mean time (seconds) of 75.66 ± 20.86 (minimum 50.88/ maximum 1200.51), females had a mean time of 84.72 ± 24.59 (minimum 56.89/ maximum 1200.51) and males 69.10 ± 14.50 (minimum 50.88/ maximum 277.58). The 200m freestyle event had an overall mean time (seconds) of 169.95 ± 41.03 (minimum 110.76/ maximum 670.73), females had a mean time of 185.11 ± 44.13 (minimum 122.35/ maximum 670.73) and males 156.55 ± 32.68 (minimum 110.76/ maximum 627.88). The 400m freestyle event had an overall mean time (seconds) of 366.73 ± 87.07 (minimum 121.91/ maximum 1028.08), females had a mean time of 395.44 ± 92.94 (minimum 133.36/ maximum 1028.08) and males 339.19 ± 70.87 (minimum 121.91/ maximum 973.56). The 800m freestyle event had an overall mean time (seconds) of 757.23 ± 180.30 (minimum 496.52/ maximum 7673.53), females had a mean time of 806.40 ± 174.77 (minimum 538.94/ maximum 2241.93) and males 707.62 ± 172.03 (minimum 496.52/ maximum 2450.40). Fig 1 presents the histograms of race times for both female and male swimmers across all freestyle swimming distances.

**Fig 2:**
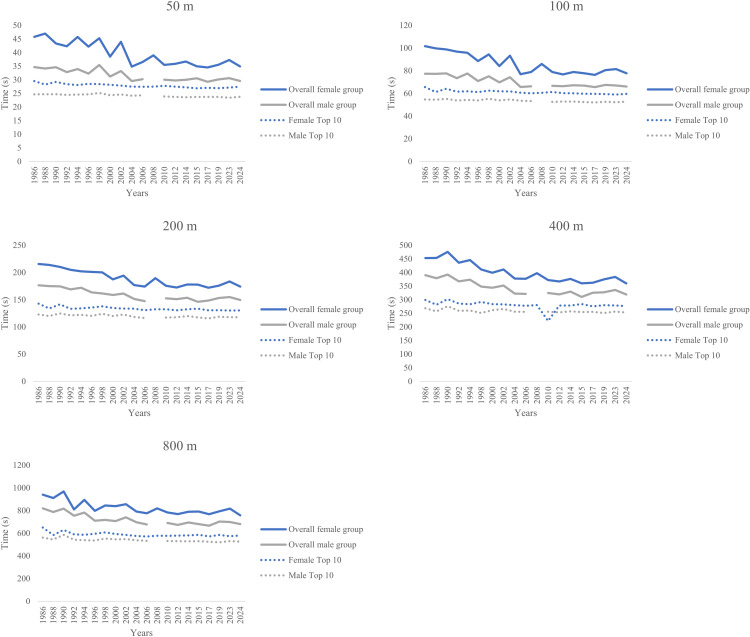
Mean times for each swimming distance by sex, including overall and top 10 performances.

**Fig 3:**
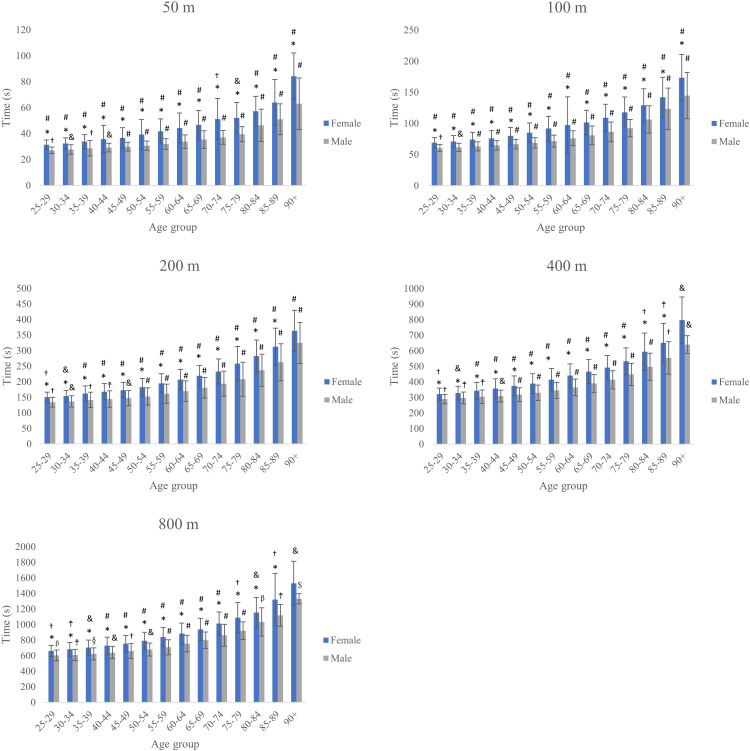
Mean race times for female and male swimmers by age group across all freestyle distances (50–800 m). **Note:** * Different from the corresponding age group of the opposite sex; # Different from all other age groups of the same sex; & Different from all age groups of the same sex, except the immediately younger one; † Different from all age groups of the same sex, except the immediately older one; ☨ Different from all age groups of the same sex, except the immediately older and younger ones; β Different from all age groups of the same sex, except the two immediately older ones; § Different from all age groups of the same sex, except the two immediately younger ones and the immediately older one; $ Different only from all younger age groups of the same sex; $ Different from all age groups of the same sex, except the two immediately younger ones.

Fig 1 presents the distribution of race times for female and male swimmers across the five freestyle distances. Generally, the histograms display unimodal distributions strongly skewed to the right (longer times), which is typical for performance time data. For each distance, the distribution for female swimmers is visibly shifted to the right compared to male swimmers, indicating generally slower times. Furthermore, as the race distance increases from 50m to 800m, the distributions for both sexes shift progressively further to the right and exhibit increased spread (variance), reflecting the longer durations and greater variability in performance over longer events.

Fig 2 illustrates the mean time for each swimming distance for both male and female participants in the overall group, as well as the mean times recorded by the top 10 athletes each year. The top 10 times for 1988 were excluded from Fig 2, as they were not included in the nationality analysis due to a lack of available information in the database. Furthermore, there was no available data for male in 2008, and 41 records had been excluded due to incomplete information.

To investigate national dominance, we employed a two-step analytical sequence. First, we descriptively assessed the frequency of top 10 appearances to address our primary hypothesis regarding quantitative contribution. As is evident from the ‘N’ column in Tables 2 and 3, the USA consistently had the highest number of swimmers in this elite cohort. Following this descriptive observation, we conducted our primary inferential analysis to determine whether the nation with the highest quantity of top swimmers also demonstrated superior performance quality, as measured by average race times. To compare the performance of countries in each swimming distance event, the five nationalities with the most participants in the top 10 times per year for each sex were selected. Other countries were classified into a single category termed “Others”. The mean time of the top 10 swimmers for each sex, year, and country were compared and are presented in Table 2 (females) and 3 (males).

**Table 2:** Differences between nationalities with female athletes in the top 10 in all freestyle distances.

Race	Ranking	Country	N	Mean (s) ± SD	CI 95%		Minimum	Maximum	χ ²	Df	p	Post-hoc differences
**Lower Limit**	Upper Limit	
**50m freestyle**	1	USA	36	27.86 ± 0.12	27.61	28.10	26.67	29.53	13.022	5	0.023	RUS vs Others*/USA*/GER*/GBR*** GBR vs Others**/JPN*/USA*
	2	GER	26	27.81 ± 0.15	27.50	28.11	26.10	28.78				
	3	RUS	17	27.35 ± 0.14	27.06	27.64	26.02	28.03				
	4	GBR	14	28.35 ± 0.28	27.75	29.95	25.73	30.05				
	5	JPN	10	27.90 ± 0.37	27.05	27.75	26.85	30.42				
	6	Others	97	27.86 ± 0.09	27.68	28.04	25.99	30.45				
**100m freestyle**	1	USA	43	61.29 ± 0.30	60.68	61.90	58.65	67.49	5.169	5	0.396	
	2	GER	21	60.68 ± 0.37	59.91	61.46	57.74	64.14				
	3	GBR	18	61.85 ± 0.56	60.67	63.03	56.96	65.93				
	4	CAN	13	60.78 ± 0.27	60.18	61.38	59.03	62.35				
	5	FRA	9	61.22 ± 0.49	60.09	62.35	59.64	64.12				
	6	Others	96	60.95 ± 0.21	60.54	61.37	56.89	67.78				
**200m freestyle**	1	USA	45	134.01 ± 0.66	132.69	135.34	126.91	149.20	10.132	5	0.072	
	2	GER	23	132.83 ± 0.70	131.38	134.27	125.76	138.09				
	3	GBR	22	136.02 ± 1.15	133.63	138.41	125.97	148.20				
	4	CAN	9	131.75 ± 0.67	130.20	133.30	129.12	135.92				
	5	FRA	8	133.36 ± 1.21	130.50	136.22	129.97	139.55				
	6	Others	93	133.46 ± 0.48	132.52	134.41	122.35	149.15				
**400m freestyle**	1	USA	45	285.50 ± 1.52	282.43	288.57	266.50	318.49	6.203	5	0.287	
	2	GER	30	275.32 ± 4.94	265.21	285.43	137.72	299.27				
	3	GBR	20	277.19 ± 7.84	260.76	293.61	136.28	320.01				
	4	FRA	9	281.92 ± 1.60	278.22	285.62	276.37	292.75				
	5	ITA	9	281.27 ± 1.07	278.80	283.74	275.19	286.45				
	6	Others	77	279.31 ± 2.96	273.40	285.21	133.36	321.35				
**800m freestyle**	1	USA	48	592.10 ± 3.43	585.19	599.00	553.49	667.59	5.468	5	0.361	
	2	GER	25	584.44 ± 3.81	576.57	592.30	547.57	621.48				
	3	GBR	21	590.42 ± 5.75	578.42	602.42	538.94	652.35				
	4	ITA	17	582.07 ± 2.54	576.69	587.45	565.74	602.96				
	5	AUS	8	615.40 ± 14.57	580.95	649.86	565.97	658.21				
	6	Others	81	587.14 ± 2.70	581.76	592.52	540.00	677.53				

Post-hoc differences are expressed as follows: * for p ≤ 0.05; ** for p ≤ 0.01; *** for p ≤ 0.001; and **** for p ≤ 0.0001.

**Table 3:** Differences between nationalities with male athletes in the top 10 in all freestyle distances.

Race	Ranking	Country	N	Mean (s) ± SD	CI 95%		Minimum	Maximum	χ ²	Df	p	Post-hoc differences
**Lower Limit**	Upper Limit	
**50m freestyle**	1	USA	35	24.42 ± 0.08	24.25	24.58	23.27	25.30	12.099	5	0.033	RUS vs GBR*/USA** Others vs USA** BRA vs USA*
	2	BRA	27	24.14 ± 0.11	23.92	24.36	23.24	25.07				
	3	GER	18	24.21 ± 0.10	24.01	24.42	23.64	25.12				
	4	RUS	13	23.92 ± 0.15	23.58	24.25	23.12	25.31				
	5	GBR	13	24.37 ± 0.16	24.02	24.71	23.73	25.52				
	6	Others	84	24.14 ± 0.07	24.00	24.28	22.94	25.47				
**100m freestyle**	1	USA	44	53.99 ± 0.18	53.62	54.35	51.56	56.60	10.164	5	0.071	
	2	BRA	22	53.53 ± 0.28	52.94	54.12	51.31	56.91				
	3	GBR	16	53.64 ± 0.35	52.89	54.39	52.05	56.65				
	4	GER	15	53.45 ± 0.32	52.77	54.13	51.75	55.95				
	5	ITA	11	53.09 ± 0.32	52.38	53.80	51.05	54.50				
	6	Others	82	53.30 ± 0.14	53.03	53.57	50.88	56.43				
**200m freestyle**	1	USA	48	120.69 ± 0.59	119.50	121.87	112.84	128.88	13.931	5	0.016	Others vs USA**/BRA**
	2	GER	19	119.55 ± 0.53	118.44	120.65	115.68	124.86				
	3	FRA	15	120.66 ± 0.67	119.22	122.10	117.75	125.24				
	4	ITA	14	119.40 ± 0.53	118.25	120.56	117.43	125.46				
	5	BRA	12	121.21 ± 0.54	120.03	122.39	118.58	125.29				
	6	Others	82	118.92 ± 0.38	118.17	119.67	110.76	128.29				
**400m freestyle**	1	USA	37	263.00 ± 1.16	260.65	265.36	248.70	280.34	25.853	5	<0.001	ITA vs USA****/BRA** Others vs USA****/BRA** FRA vs USA** GER vs USA*
	2	ITA	23	256.07 ± 1.33	253.30	258.84	244.17	269.99				
	3	GER	17	258.75 ± 1.27	256.05	261.44	250.78	269.06				
	4	FRA	12	257.93 ± 1.53	254.57	261.30	253.50	272.88				
	5	BRA	9	263.65 ± 2.55	257.78	269.52	256.97	276.70				
	6	Others	82	256.04 ± 1.90	252.26	259.81	121.91	283.30				
**800m freestyle**	1	USA	36	545.77 ± 2.76	540.16	551.38	516.88	579.80	32.914	5	<0.000	ESP vs Others*/GER*/USA****/BRA**** ITA vs Others*/GER*/USA***/BRA**** Others vs USA**/BRA*** GER vs BRA*
	2	ITA	30	531.29 ± 2.39	526.40	536.19	508.77	570.22				
	3	GER	21	538.91 ± 2.84	533.00	544.82	515.45	562.57				
	4	BRA	12	564.76 ± 7.75	547.72	581.81	522.85	605.84				
	5	ESP	10	527.00 ± 2.51	521.32	532.68	518.86	542.93				
	6	Others	81	537.46 ± 1.96	533.57	514.36	496.52	595.55				

Post-hoc differences are expressed as follows: * for p ≤ 0.05; ** for p ≤ 0.01; *** for p ≤ 0.001; and **** for p ≤ 0.0001.

Analysis of the top 10 male performers by nationality (Table 3) revealed significant differences between countries across several freestyle distances. In the 50m freestyle, while multiple nations showed competitive mean times, post-hoc tests indicated that swimmers from the USA (mean 24.42 s) were significantly slower than those from Russia (mean 23.92 s, p ≤ 0.05) and Brazil (mean 24.14 s, p ≤ 0.05). No significant differences between the top nations were found in the 100m freestyle. For the 200m event, swimmers categorized as ‘Others’ (mean 118.92 s) and from Germany (mean 119.55 s) showed significantly faster times than those from the USA (mean 120.69 s, p ≤ 0.01) and Brazil (mean 121.21 s, p ≤ 0.01). The differences became more pronounced in longer distances. In the 400m freestyle, swimmers from Italy (mean 256.07 s), ‘Others’ (mean 256.04 s), France (mean 257.93 s), and Germany (mean 258.75 s) all performed significantly faster than swimmers from the USA (mean 263.00 s, p ≤ 0.01 to p < 0.001) and Brazil (mean 263.65 s, p ≤ 0.01). Similarly, in the 800m freestyle, significant differences were widespread, with Italian (mean 531.29 s), Spanish (mean 527.00 s), ‘Others’ (mean 537.46 s), and German (mean 538.91 s) swimmers significantly outperforming those from the USA (mean 545.77 s, p ≤ 0.01 to p < 0.0001) and Brazil (mean 564.76 s, p < 0.0001). These findings highlight the variable national dominance across different freestyle distances for elite male master swimmers.

The generalized linear models’ findings show that age group, sex, and the interaction between sex and age group all had significant effects on the 50m, 100m, 200m, 400m, and 800m races. Specifically, for the 50m races, the effect of sex was significant (χ^2^ (1) = 3451.941, p < 0.001), as was the effect of age group (χ^2^ (13) = 19,295.169, p < 0.001), and the interaction between sex and age group (χ^2^ (13) = 654.671, p < 0.001). The model’s quality was evaluated through the Omnibus test (p < 0.001) and the Akaike Information Criterion (AIC = 155032.192). In the 100m races, significant effects were noted for sex (χ^2^ (1) = 2377.383, p < 0.001), age group (χ^2^ (13) = 22158.355, p < 0.001), and the interaction between sex and age group (χ^2^ (13) = 418.346, p < 0.001). The model demonstrated superiority over the null model as indicated by the Omnibus test (p < 0.001), with its quality evaluated through the AIC (AIC = 157742.859). In the 200m races, significant effects were observed for sex (χ^2^ (1) = 1501.899, p < 0.001), age group (χ^2^ (13) = 18395.753, p < 0.001), and the interaction between sex and age group (χ^2^ (13) = 184.003, p = 0.001). The model demonstrated superiority over the null model as indicated by the Omnibus test (p < 0.001), with its quality evaluated through the AIC (AIC = 128567.078). For the 400m races, sex (χ ^2^ (1) = 791.539, p < 0.001), age group (χ ^2^ (13) = 11216.661, p < 0.001), and the interaction between sex and age group (χ ^2^ (13) = 125.969, p = 0.001) all had significant effects. The model’s quality was evaluated using the AIC (AIC = 117328.260), and the Omnibus test showed that it was once again superior to the null model (p < 0.001). Finally, for the 800m races, sex (χ ^2^ (1) = 493.796, p < 0.001), age group (χ ^2^ (13) = 11747.060, p < 0.001), and the interaction between sex and age group (χ ^2^ (13) = 80.054, p = 0.001) all had significant effects. The model was again superior to the null model according to the Omnibus test (p < 0.001), and its quality was assessed using the AIC (AIC = 123578.530).

Fig 3 illustrates how mean race times increase with advancing age across all freestyle distances and highlights the performance gap between male and female master swimmers. The curves for each event rise progressively from the 25–29 to the 90 + age groups, indicating a decline in speed with age. This age-related slowdown is more pronounced in longer events: the increment in mean time between consecutive age brackets is smallest in the 50m and largest in the 800m. At every age group and distance, the female curve lies above the male curve, confirming that women record slower mean times than men; this gap is relatively narrow in early age groups but widens gradually – particularly beyond age 60 – before showing a slight leveling off in the oldest categories.

## Discussion

The purpose of this study was to explore the trends in participation and performance by nationality among age group freestyle swimmers in World Aquatics competitions for the 50m, 100m, 200m, 400m and 800m events held between 1986 and 2024. Our hypotheses were (i) the USA would demonstrate quantitative dominance by contributing the greatest number of top 10 performers, and we subsequently sought to determine if this was matched by qualitative dominance in the form of superior average performance times, (ii) males would have better times than females in the same age group, and (iii) the number of male and female participating in long distances tend to equalize. The main findings of this study support these hypotheses: Firstly, the evidence overwhelmingly indicates that across all distances, the USA contributed the highest number of finishers in the top 10 fastest times each year, indicating depth of elite performance – even in events where the absolute fastest average time was recorded by another country. The Kruskal–Wallis tests and subsequent Bonferroni‐corrected post-hoc comparisons show that the USA’s mean top 10 times are significantly lower than those of most other nations across multiple distances. In the women’s 50m freestyle, Russia recorded the quickest mean top 10 time (27.35s) versus 27.86s for the USA; in the 100m, Germany led with 60.68s compared to the USA’s 61.29s; in the 200 m, Canada held the fastest average (131.75s) against the USA’s 134.01s. Likewise, in the 400m and 800m events, Germany (275.32s) and Italy (582.07s) topped the respective top 10 averages, surpassing the USA’s 285.50s and 592.10s. These results indicate that other nations frequently outpace the USA in mean performance times, even as the US remains unrivaled in depth of participation.

While the USA contributed the greatest number of top 10 swimmers across all five freestyle distances, the absolute fastest average times within those top 10 groups did not consistently belong to American athletes. For instance, in the men’s 50m freestyle, both Russia (23.92s) and Brazil (24.14s) outperformed the USA’s 24.42s. Similarly, in the 100m event, Italy led with a top 10 mean of 53.09s, ahead of the USA’s 53.99s. In the 200m, swimmers categorized as ‘Others’ posted the quickest average (118.92s), and they also edged out the USA in the 400m (256.0 s vs. USA’s 263.00s). Finally, in the 800m freestyle, Spain claimed the fastest mean time (527.00s), compared to 545.77s for the USA. These findings underscore that, although the USA supplied the greatest number of top 10 finalists, other nations and aggregated ‘Others’ have captured the outright fastest top 10 times at each distance. While Brazilian male swimmers show strong performances in the 50m and 100m freestyle events, in the longer distances, such as the 200m, 400m, and 800m freestyle, the dominance shifts slightly, with countries like Italy, France, Great Britain, and Germany making notable appearances in the top ranks.

### USA leading in producing elite freestyle swimmers

The findings support the study’s hypothesis (i) that the US regularly generates top 10 swimmers. Considering its past swimming dominance, the United States’ superiority makes sense. The long-term success of American swimmers may be attributed to elements like strong training regimens, infrastructure, and talent development [23,24]. For instance, in the United States, U.S. Masters Swimming (USMS) provide a wide range of resources for swimmers, such as a vast library of workouts for various training styles (https://www.usms.org/). Similarly, other countries listed in the top ten are also actively promoting master swimming and swimming infrastructure: the German Swimming Federation (Deutscher Schwimm-Verband or DSV) provides an extensive directory of swimming clubs and competitions for age group swimmers (https://www.dsv.de/home/; https://www.dsv.de/masterssport/). Similarly, Swim England Masters in Great Britain offers information on various races, links to the masters’ community, as well as guidance on technique and nutrition (https://www.swimming.org/masters/). However, the Brazilian Confederation of Aquatic Sports (Confederação Brasileira de Desportos Aquáticos/CBDA) focuses pool swimming, open-water swimming, artistic swimming, water polo, and diving. In contrast, master swimming is a lower priority (https://www.cbda.org.br/). These regulations are crucial in providing swimmers with easy access to pools, which raises the quantity of workouts and swimmers’ performance in these nations. Consequently, one factor contributing to these countries’ success may be their excellent swimming facilities [26].

### Male swimmers achieve faster times than their female counterparts

Secondly – and accordingly to our hypothesis (ii) – sex differences in mean times were observed across all distances, with males generally exhibiting typically faster mean race times than female. For instance, in the 50m freestyle, the mean time for males is 24.42 seconds, while for females, it is 27.86 seconds, indicating that males swim significantly faster. This pattern is consistent across all distances. In the 100m freestyle, males have a mean time of 52.64 seconds compared to females’ 61.08 seconds. The difference is even more pronounced in the 800m freestyle, where males have a mean time of 545.77 seconds, significantly faster than the 592.10 seconds for females. The statistical significance of these differences is supported by the p-values in the analysis, which consistently indicate that sex is a significant factor in swimming performance across all distances. The significant effect of sex across all distances in this study is consistent with findings by Knechtle et al. [8], who reported that male swimmers tend to maintain faster times across all age groups in master swimming competitions.

This sex gap in performance highlights the physiological distinctions between male and female athletes and is consistent with earlier studies in competitive swimming [16,42,43,44,8]. These differences have been extensively studied in sports science. They can be attributed to various physiological factors: Since males typically have a higher proportion of lean muscle mass and lower body fat percentage than females, the difference in body composition allows male to generate more strength and power [28,45,46]. Studies have shown that muscle mass and strength positively correlate with swimming performance [27,47]. Research further has demonstrated that higher testosterone levels in males contribute to enhanced muscular strength and power, which can translate into faster swimming times [48,49]. Further reasons for sex differences that affect peak performance are anatomical characteristics, such as males tending to have larger lung capacities, broader shoulders, and longer limbs compared to females [16,50,51]. These anatomical differences provide advantages in swimming efficiency, propulsion, and oxygen uptake, all of which are critical for achieving faster race times, specifically when it comes to freestyle swimming [52]. While both male and female swimmers strive to optimize their stroke efficiency in freestyle swimming, variations in biomechanical factors – such as differences in swimming technique and stroke mechanics between sexes – may contribute to the observed performance gap and influence performance outcomes [53,54,51].

Age was also a significant factor, with performance declining progressively as age increased, particularly in the longer distances. The decline in performance has been extensively recorded in scientific literature. It is generally attributed to age-related physiological alterations, including diminished muscle mass, reduced aerobic capacity, and prolonged recovery periods [55]. An analysis of male swimmers aged 50–90 years participating in the 200-m freestyle events of the World Master Championships showed that, compared to the younger groups, the older groups had smaller stroke lengths and higher stroke frequency [56]. The interaction between sex and age group also suggests that the performance loss rate varies between male and female, with males generally experiencing a more gradual drop [55]. Concerning participation, the findings in this study are in line with similar analyses in other sports, where at longer distances, the number of female and male participants trend to equalize [37]. This pattern mirrors aging‐related performance trajectories observed across a range of master sports disciplines [57].

In addition, interesting observations concerned the performance-related trends across calendar years (Fig 1). Progression and variability in elite swimming have been documented in Olympic cohorts [58]. This analysis showed that both sexes improved performance across calendar years; however, these changes were more remarkable in the overall sample than in top 10 swimmers. An improvement in swimming performance over 10 years (2013–2022) was seen in all swimming events within a 50-m pool, considering the top 200 world-ranked entries, too [59]. Nevertheless, our analysis of the top 10 swimmers indicated little progress in their performance, reflecting low progress in high-performance science and practice during the last years. Instead, the non-elite swimmers seemed to benefit from the ongoing increased participation of master athletes in swimming events [8]. Master athletes are also extending the limits of human endurance [51]. This observation was in agreement with the research of Akkari et al. [60], who reported that master athletes improved their athletic performance over the years, in contrast to younger athletes, whose performance stagnated.

The study also has some limitations. Firstly, the data used only spans the previous 40 years and focuses on the five nationalities with the most top 10 swimmer appearances (plus all other nations grouped together); therefore, the methodological approach limits the generalizability of these findings. Different results could be achieved by including all countries instead of this selected subset. Secondly, the study excludes other potential contributing elements, including training regimens, coaching tactics, and physiological features, and only concentrates on age groups, sex, and nationality. Similarly, focusing the analysis solely on the top 10 fastest times per year might have excluded strong performers or nations consistently ranking just outside this elite cutoff, potentially limiting the full scope of national performance representation. Given the complexity of swimming’s elite performance, a more complete collection of variables should be considered for thorough analysis. Nonetheless, using statistical models verified by the Omnibus test and AIC strengthens the validity of the study’s findings. Furthermore, while statistical significance was assessed, the absence of reported effect sizes limits the interpretation regarding the practical magnitude of some observed differences, particularly given the large sample sizes involved in parts of the analysis.

## Conclusion

This study investigated participation and performance trends among age group freestyle swimmers by nationality in World Aquatics competitions from 1986 to 2024. The main findings supported our hypotheses that (i) the USA demonstrated clear quantitative dominance – contributing the greatest number of top 10 performers – although this was not always matched by superior average times; (ii) males consistently outperformed females within each age category; and (iii) female participation numbers approached parity with males in the longer distances. Our data confirm that, while the USA leads in depth of elite participation, other nations frequently record the fastest mean times in specific events. Countries such as Germany, Great Britain, Brazil, and Italy appear prominently among top performers in certain distances, underscoring a broadening of the elite master-swimming landscape. Across all distances, a significant sex-based performance gap persists, with males registering faster race times than females in every age group. Moreover, participation ratios indicate that female swimmers increasingly match male swimmer numbers in the 400m and 800m events. While our study did not assess physiological or training variables directly, these findings establish a robust framework for future investigations into the determinants of both quantitative depth and qualitative excellence across nations and sexes in master’s freestyle swimming. Future research will be facilitated by the in-depth comparisons and nationality categories provided by the study, which contribute to a better knowledge of swimming performance worldwide.
